# IL-8, GRO and MCP-1 produced by hepatocellular carcinoma microenvironment determine the migratory capacity of human bone marrow-derived mesenchymal stromal cells without affecting tumor aggressiveness

**DOI:** 10.18632/oncotarget.10288

**Published:** 2016-06-25

**Authors:** Juan Bayo, Alejandrina Real, Esteban J. Fiore, Mariana Malvicini, Leonardo Sganga, Marcela Bolontrade, Oscar Andriani, Carolina Bizama, Cristóbal Fresno, Osvaldo Podhajcer, Elmer Fernandez, Manuel Gidekel, Guillermo D. Mazzolini, Mariana G. García

**Affiliations:** ^1^ Gene Therapy Laboratory, Instituto de Investigaciones en Medicina Traslacional, Facultad de Ciencias Biomédicas, CONICET, Universidad Austral, Buenos Aires, Argentina; ^2^ Stem Cells Laboratory, IBYME, CONICET, Buenos Aires, Argentina; ^3^ Liver Unit, Hospital Universitario Austral, Derqui-Pilar, Argentina; ^4^ Universidad Católica, Santiago, Chile; ^5^ BioScience Data Mining Group, Catholic University of Córdoba, Córdoba, Argentina; ^6^ Fundación Instituto Leloir, CONICET, Buenos Aires, Argentina; ^7^ Universidad de la Frontera, Temuco, Chile; ^8^ Universidad Autónoma de Chile, Santiago, Chile

**Keywords:** human mesenchymal stromal cells, tumor microenvironment, IL-8, human hepatocellular carcinoma, migration

## Abstract

New therapies are needed for advanced hepatocellular carcinoma (HCC) and the use of mesenchymal stromal cells (MSCs) carrying therapeutic genes is a promising strategy. HCC produce cytokines recruiting MSCs to the tumor milieu and modifying its biological properties. Our aim was to study changes generated on human MSCs exposed to conditioned media (CM) derived from human HCC fresh samples and xenografts. All CM shared similar cytokines expression pattern including CXCL1-2-3/GRO, CCL2/MCP-1 and CXCL8/IL-8 being the latter with the highest concentration. Neutralizing and knockdown experiments of CCL2/MCP-1, CXCL8/IL-8, CXCR1 and CXCR2 reduced *in vitro* MSC migration of ≥20%. Simultaneous CXCR1 and CXCR2 neutralization resulted in 50% of MSC migration inhibition. MSC stimulated with CM (sMSC) from HuH7 or HC-PT-5 showed a 2-fold increase of migration towards the CM compared with unstimulated MSC (usMSC). Gene expression profile of sMSC showed ~500 genes differentially expressed compared with usMSC, being 46 genes related with cell migration and invasion. sMSC increased fibroblasts and endothelial cells chemotaxis. Finally, sMSC with HuH7 CM and then inoculated in HCC tumor bearing-mice did not modify tumor growth. In this work we characterized factors produced by HCC responsible for the changes in MSC chemotactic capacity with would have an impact on therapeutic use of MSCs for human HCC.

## INTRODUCTION

Hepatocellular carcinoma (HCC) is the 2^nd^ cause of cancer-related death worldwide and its incidence is steadily increasing despite the advances in the understanding of hepatocarcinogenesis [1]. The vast majority of HCC arise in livers with underlying cirrhosis due to chronic hepatitis B or C virus infection or heavy alcohol consumption [2]. Unfortunately, most patients with HCC are diagnosed at advanced stages when curative options such as liver resection, transplantation or radiofrequency ablation are not feasible. Sorafenib, a multikinase inhibitor drug, is the standard of care for patients with advanced HCC, however, median overall survival was 10.7 months in the sorafenib group and 7.9 months in the placebo group [3]. Therefore, development of new therapeutic approaches is required for HCC. In this scenario, cells armed with antitumor genes or carrying oncolytic viruses have been be proposed as therapeutic tools in several preclinical models [4]. In particular, mesenchymal stromal cells (MSCs) have shown the capability to migrate to tumors *in vivo* making them a promising therapeutic strategy in cancer therapy.

MSCs are a heterogeneous population of multipotent cells present in almost all adult tissues that migrate to sites of injury and have the capability to differentiate into mesodermal derivatives (adipocytes, osteoblasts and chondroblasts) [5]. In addition, MSCs have also shown immunoregulatory and pro-regenerative effects due to the secretion of several growth factors and cytokines [6]. MSCs are usually isolated from bone marrow (BM), adipose tissue or from neonatal tissues such as umbilical cord [7]. Tumors have been considered as unresolved wounds since a continuous process of damage and repair is taking place [8]. At least in part, this process is orchestrated by an extensive crosstalk between cancer cells and its microenvironment. In this context, tumor tropism of MSCs make them a potential tool for the development of new cancer therapies as carriers of oncolytic vectors or producing antitumor genes [4]. However, mechanisms and signals involved in their recruitment to the tumor are not completely elucidated. It is known that MSCs express several cytokines and chemokines receptors allowing their migration in response to signals released by the tumor and their microenvironment. It has been proposed that MSCs use different combination of cytokines and growth factors for their homing depending on the tumor type [9]. Results obtained from *in vitro* and *in vivo* studies identified key factors involved in MSC migration such as VEGF, PDGF, TGF-β, CCL2/MCP-1, CXCL8/IL-8, TNF-α, IL-1β, IL-6, CXCL12/SDF-1 or HGF [10, 11]. Particularly, we have recently shown that MSCs migrate towards HCC, partially through the autocrine motility factor (AMF)/autocrine motility factor receptor (AMFR) [12]. In addition, MSCs were isolated from different tumors types including HCC and HCC-associated MSCs have shown pro-tumorigenic properties demonstrating that these cells could be educated by the tumor [13]. Therefore, several aspects of MSC homing into tumors should be addressed before these cells can be considered for clinical purposes, including the evaluation of which cytokine and/or growth factors are released by HCC and how these factors affect MSC migration and its interaction with the different components of the entire tumor.

Here, for the first time, we report the identification of CXCL8/IL-8, CCL2/MCP-1, and CXCL1-2-3/GRO as chemotactic axis for MSC migration toward human HCC. We further demonstrate that HCC-stimulated MSCs increased their chemotactic potential and modified their gene profile pattern including genes involved in migration and invasion process. In addition, we observed that stimulated MSCs secreted several chemokines that induce the recruitment of fibroblasts, endothelial cells and peripheral blood mononuclear cells (PBMNCs) towards the HCC. Systemic administration of stimulated or unstimulated MSCs did not affect HCC aggressiveness *in vivo*.

## RESULTS

### Hepatocellular carcinoma-derived factors induce human MSC migration

**Figure 1:**
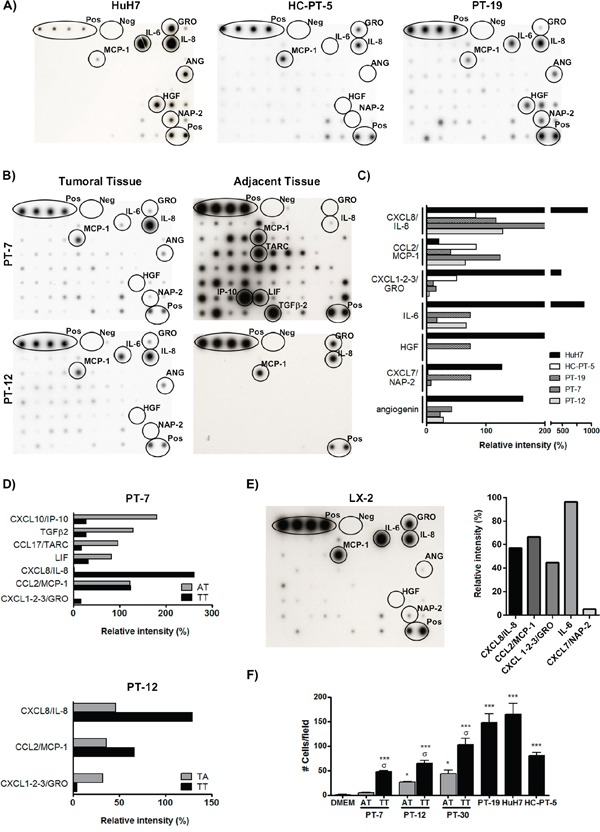
HCC factors induce MSC *in vitro* chemotaxis **A.** Antibody array of CM derived from subcutaneous (s.c.) tumors generated in nude mice using HuH7 or HC-PT-5 cells, or fresh HCC patient sample (PT-19). Pos: positive control; Neg: negative control. **B.** Antibody array of CM derived from fresh tumor tissue or non-tumoral liver parenchyma (patient samples PT-7 or PT-12). **C.** Quantification of the antibody array signal from tumoral CM by densitometry. Results are expressed as relative intensity (%); positive controls were used to normalize the results. **D.** Quantification of antibody array signal and comparison between factors present in tumoral tissue (TT) and non-tumoral liver parenchyma (AT) from PT-7 (upper panel) and PT-12 (bottom panel). Results are expressed as relative intensity (%). **E.** Antibody array of CM derived from LX-2 cell line (left panel) and its quantification by densitometry (right panel). Results are expressed as relative intensity (%) and positive controls were used to normalize the results. **F.** MSC *in vitro* chemotaxis was analyzed with a modified Boyden chamber assay using the previously analyzed CM as chemoattractant. Results are expressed as number of cells per field ± SEM from 3 independent experiments, each performed in quadruplicate. *p<0.05 and ***p<0.01 vs DMEM; ^σ^p<0.05 vs AT from the same patient sample (ANOVA and Dunn's post test).

We and others have demonstrated that MSCs migrate *in vitro* and *in vivo* towards HCC [14]. Recently, we reported that MSC *in vitro* migration toward HCC is mediated in part by AMF [12], however other cytokines and growth factors could also be involved. In order to identify factors that could mediate MSC recruitment to human HCC we examined the cytokine profile of different human HCC samples. Conditioned medium (CM) derived from fresh HCC patient samples (PT-7, PT-12 and PT-19) or from tumors induced by the inoculation of a primary culture from a HCC patient (HC-PT-5) or HuH7 HCC cells in nude mice were analyzed with a cytokine antibody array (Figure 1A and 1B). Quantification of arrays showed that all CM analyzed contain CXCL1-2-3/GRO, CCL2/MCP-1, and CXCL8/IL-8 being the latter cytokine the most important (Figure 1C). Interestingly, CM derived from HuH7, PT-7, PT-12 and PT-19 showed high levels of IL-6 and angiogenin while the CM from the HC-PT-5 tumor did not present these cytokines. Regarding CXCL7/NAP-2 and HGF only were found in CM from HuH7 and PT-19. We next wondered if these factors are specific for the tumoral tissue (TT) or if they can be found in the adjacent non-tumoral liver parenchyma (AT) derived from the same patients with HCC; primary biliary cirrhosis (PT-7) or cryptogenic cirrhosis (PT-12). Cytokine antibody arrays of AT showed that the 2 samples have a different pattern of cytokine expression. On one hand, CM derived from AT-PT-7 showed higher levels of CXCL10/IP-10, TGFβ2, CCL17/TARC and LIF but lower levels of CXCL8/IL-8 and CXCL1-2-3/GRO in comparison with TT (Figure 1B and 1D). On the other hand, CM from AT-PT-12 presented the expression of few factors compared to the other CM. Only CXCL8/IL-8, CCL2/MCP-1 and CXCL1-2-3/GRO were found (Figure 1B and 1D). It should be noted that levels of CXCL8/IL-8 and CCL2/MCP-1 were lower than those observed for the CM derived from the TT but the expression of CXCL1-2-3/GRO was higher (Figure 1D). The cytokine pattern was also evaluated in hepatic stellate cells (HSC), the main cell type involved in hepatic fibrogenesis. Cytokines present in the CM derived from the HSC cell line LX-2 were analyzed. As a result, we observed an expression pattern similar to AT-PT-12; in addition, IL-6 and CXCL7/NAP-2 were also detected (Figure 1E). Then, we assessed if these differences affected the MSC *in vitro* chemotaxis capability. *In vitro* migration assays showed that MSCs migrate preferentially to tumor derived CM (Figure 1F). However, CM derived from AT-PT-12 and AT-PT-30 were able to induce MSC migration in some degree.

### CXCL8/IL-8, CXCL1-2-3/GRO and CCL2/MCP-1 axes are important for MSC *in vitro* migration towards human HCC

**Figure 2:**
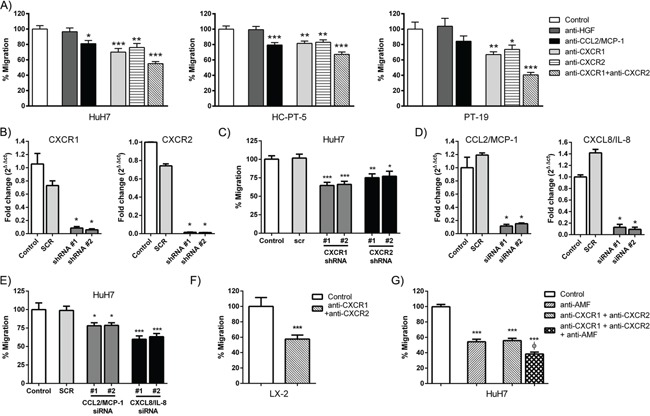
CXCL8/IL-8, CXCL1-2-3/GRO and CCL2/MCP-1 axes are important for MSC *in vitro* migration **A.** *In vitro* chemotaxis assay of MSCs towards CM derived from HuH7 (left panel), HC-PT-5 (middle panel) or PT-19 (right panel). Pre-treatment with neutralizing antibodies to inhibit HGF or CCL2/MCP-1 was performed in each CM, while inhibition of CXCR1, CXCR2 or CXCR1 + CXCR2 was performed in MSCs. Results are expressed as percentage of control (isotype control IgG) ± SEM. *p<0.05, **p<0.01 and ***p<0.001 vs control (ANOVA and Dunnett's test). **B.** qRT-PCR analysis of CXCR1 or CXCR2 expression in MSCs 72 h after transduction with 2 different specific shRNAs retrovirus. Untransduced MSCs (control) and scrambled negative control (SCR) were used. *p<0.05 vs control and SCR (Kruskal-Wallis test). **C.** *In vitro* chemotaxis assay of CXCR1 or CXCR2 depleted MSCs towards CM derived from HuH7. Results are expressed as percentage of control (untransduced MSCs) ± SEM. *p<0.05, **p<0.01 and ***p<0.001 vs control and SCR (ANOVA and Dunnett's test). **D.** qRT-PCR analysis of CCL2/MCP-1 or CXCL8/IL-8 expression in HuH7 72 h after transfection with specific siRNAs. Untransfected HuH7 and scrambled negative control (SCR) were used. *p<0.05 vs control and SCR (Kruskal-Wallis test). **E.** *In vitro* chemotaxis assay of MSCs towards CM derived from monolayer of CCL2/MCP-1 or CXCL8/IL-8 depleted HuH7 cells. Results are expressed as percentage of control (untransfected MSCs) ± SEM. *p<0.05 and ***p<0.001 vs control and SCR (ANOVA and Dunnett's test). **F.** *In vitro* chemotaxis assay of MSCs towards CM derived from LX-2 cells. Pre-treatment with neutralizing antibodies to inhibit CXCR1 + CXCR2 was performed in MSCs. Results are expressed as percentage of control (isotype control IgG) ± SEM. ***p<0.001 vs control (t-test). **G.** *In vitro* chemotaxis assay of MSCs towards CM derived from HuH7. Pre-treatment with neutralizing antibody to inhibit AMF was performed in the CM, while antibody inhibition of CXCR1 + CXCR2 was performed in MSCs. Results are expressed as percentage of control (isotype control IgG) ± SEM. ***p<0.001 vs control; ^Ф^p<0.05 vs anti-AMF and anti-CXCR1+anti-CXCR2 (ANOVA and Dunnett's test). All of the above data represent the average of 3 independent experiments, each performed in quadruplicate.

CXCL8/IL-8, CXCL1-2-3/GRO and CCL2/MCP-1 have been described as chemotactic factors for MSCs. In addition these cytokines were found in all the CM from human HCC samples. Therefore, we hypothesized that these chemokines could be involved in MSC chemotaxis toward HCC. Neutralizing antibody against CCL2/MCP-1 reduced the capability of inducing MSC chemotaxis toward CM derived from the human HCC tumors HuH7, HC-PT-5 or PT-19 by around a 20% while the anti-HGF antibody did not (Figure 2A). In a similar way incubation of MSCs with anti-CXCR1 or anti-CXCR2 antibodies reduced their *in vitro* chemotaxis capability to HCC by around a 20~30%. Furthermore, when both receptors were neutralized together, MSC chemotaxis was reduced by around 50~60% (Figure 2A). The migration involving CXCR1 and CXCR2 was also evaluated by knocking down their expression in MSCs by shRNA. Two-different shRNAs were used for each receptor (Figure 2B). Then, migratory experiments showed decreased MSC migration of around 20~30% towards HuH7 CM compared to control (SCR) (Figure 2C). To further validate these migratory axes in MSC migration, CCL2/MCP-1 and CXCL8/IL-8 production was inhibited in HuH7 by siRNA. Two different siRNA were used for knockdown of each cytokine (Figure 2D). Chemotaxis experiments showed decreased migration of MSCs towards the HuH7 CM after CCL2/MCP-1 or CXCL8/IL-8 cytokine inhibition (Figure 2E). Then, considering the key role of the HSC cells in liver cirrhosis and hepatocarcinogenesis, the CXCR1 and CXCR2 axes were studied in MSC migration to LX-2 CM. Similarly to HCC CM samples, MSC chemotaxis towards LX-2 CM was decreased when CXCR1 and CXCR2 were blocked (Figure 2F). In our previous work [12] we demonstrated that the blockage of AMF decreased MSC migration towards HCC CM. To further analyze the cooperative role of several cytokines in the recruitment of MSCs to HCC, neutralizing experiments were performed. The simultaneous blockage of AMF, CXCR1 and CXCR2 resulted in a higher decrease of MSC migration in comparison with AMF alone or with CXCR1 + CXCR2 (Figure 2G).

### Exposure of MSCs to human HCC conditioned media enhanced their migratory capacity

**Figure 3:**
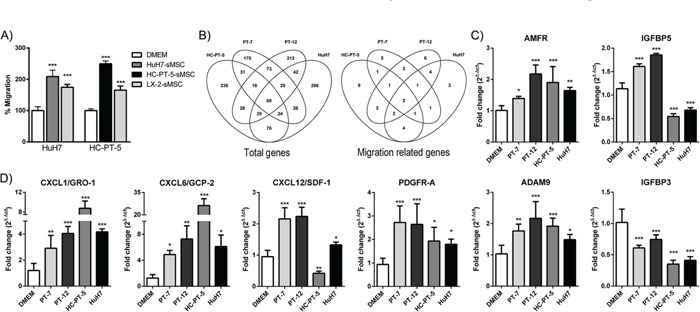
HCC CM enhanced MSC migratory capacity and induced changes in their gene expression pattern **A.** Stimulation of MSCs with CM of HuH7 (dark grey bar), HC-PT-5 (black bar) or LX-2 (grey bar) increases chemotaxis towards CM derived from HuH7 or HC-PT-5 cells compared to unstimulated MSCs (DMEM, white bars). Results are expressed as percentage of control (DMEM) ± SEM of 3 independent experiments, each performed in quadruplicate. ***p<0.001 vs unstimulated cells (DMEM, ANOVA and Tukey's comparison test). **B.** Venn diagram showing overlap of all the transcripts (left panel) or the genes related with cell migration and invasion (right panel) modulated in MSCs after the exposure to the different CM (HC-PT-5, PT-7, PT-12 or HuH7). **C-D.** q-RT-PCR validation of certain genes related with cell migration and invasion in MSCs stimulated with the CM (PT-7, PT-12, HC-PT-5 or HuH7) or unstimulated MSCs (DMEM). *p<0.05, **p<0.01 and ***p<0.001 vs unstimulated MSCs (DMEM, ANOVA Dunnett's test).

Taking into account that HCC released factors were able to induce MSC migration we investigated whether this capability could be enhanced by the exposure to the CM. With this aim, MSCs were incubated for 24 hours in the presence of the HuH7 or the HC-PT-5 CM. Migration assay showed a 2-fold increase in the migration of stimulated MSCs toward the CM derived from the HuH7 or HC-PT-5 tumors in comparison with unstimulated MSCs (Figure 3A). Similarly, stimulation of MSCs with LX-2 CM also increased their migration (Figure 3A). With the aim of identifying the molecular pathways involved in this chemotactic effect, gene expression analysis of MSCs exposed to the CM derived from the HCC tumors (PT-7, PT-12, HuH7 and HC-PT-5) was performed using cDNA microarray. Gene expression profile showed that MSCs stimulated with CM from PT-7 differentially expressed 445 genes in comparison with unstimulated cells. In a similar way, 511, 521 and 511 genes were differentially expressed after the exposure to CM derived from PT-12, HC-PT-5 or HuH7, respectively. Overlap of transcripts modulated after the exposure to the different CM is shown in a Venn diagram in Figure 3B. Among them 46 genes related with cell migration and invasion toward tumors were identified. The overlap of the transcripts of these selected genes in the MSCs exposed to the different CM was depicted with a Venn diagram in Figure 3B. In particular, we found cytokines, receptors, adhesion molecules and proteases that modulate the extracellular matrix and their interaction with the cell receptors (described in Table 1). Interestingly, AMFR and IGFBP5 were modulated after the exposure to all the CM. However, validation by qPCR analysis showed not only these genes modulation after MSC exposure to the tumoral CM (Figure 3C) but also the modulation of CXCL1/GRO-1, CXCL6/GCP-2, CXCL12/SDF-1, PDGFR-A, ADAM9 and IGFBP3 (Figure 3D).

**Table 1:** Genes differentially expressed in MSCs stimulated with CM (PT-7, PT-12, HC-PT-5 or HuH7) in comparison to unstimulated MSCs

	Gene	Gene ID	P value	RE	P value	RE	P value	RE	P value	RE
**CM**			**PT-7**		**PT-12**		**HC-PT-5**		**HuH7**	
Cytokines	**CTGF**	1490	0.01	1.31	0.004	1.37	n.c.	n.c.	n.c.	n.c.
	**CYR61**	3491	<0.001	1.78	<0.001	1.70	n.c.	n.c.	n.c.	n.c.
	**TNFSF12**	8742	n.c.	n.c.	0.01	1.46	n.c.	n.c.	0.046	1.32
	**CXCL1**	2919	n.c.	n.c.	n.c.	n.c.	0.006	1.97	0.012	1.85
	**LIF**	3976	n.c.	n.c.	0.01	1.38	<0.001	1.60	0.001	1.54
	**CSF1**	1435	0.011	1.36	n.c.	n.c.	n.c.	n.c.	n.c.	n.c.
	**MIF**	4282	0.035	1.15	n.c.	n.c.	n.c.	n.c.	n.c.	n.c.
	**PDGFC**	56034	0.049	1.18	n.c.	n.c.	n.c.	n.c.	n.c.	n.c.
	**CXCL6**	6372	n.c.	n.c.	n.c.	n.c.	0.044	1.35	n.c.	n.c.
	**IL6**	3569	n.c.	n.c.	n.c.	n.c.	0.024	1.43	n.c.	n.c.
	**CXCL14**	9547	n.c.	n.c.	<0.001	0.56	n.c.	n.c.	0.014	0.74
	**CXCL12**	6387	n.c.	n.c.	n.c.	n.c.	0.003	0.72	n.c.	n.c.
Receptors	**AMFR**	267	0.024	1.24	0.022	1.25	<0.001	1.46	0.005	1.34
	**TRAF7**	84231	0.039	1.35	n.c.	n.c.	n.c.	n.c.	0.029	1.37
	**PDGFRA**	5156	n.c.	n.c.	n.c.	n.c.	0.036	1.36	0.032	1.37
	**IGF1R**	3480	n.c.	n.c.	0.002	1.86	n.c.	n.c.	n.c.	n.c.
	**TNFRSF11B**	4982	n.c.	n.c.	0.006	1.48	n.c.	n.c.	n.c.	n.c.
	**GPR108**	56927	n.c.	n.c.	n.c.	n.c.	n.c.	n.c.	0.043	1.39
	**CD74**	972	n.c.	n.c.	0.042	0.79	n.c.	n.c.	0.007	0.72
Proteases	**FURIN**	5045	0.019	1.32	0.005	1.42	n.c.	n.c.	0.028	1.29
	**ADAMTS1**	9510	0.002	1.46	0.002	1.47	n.c.	n.c.	n.c.	n.c.
	**MME**	4311	0.009	1.26	n.c.	n.c.	0.006	1.28	n.c.	n.c.
	**FAP**	2191	n.c.	n.c.	<0.001	1.51	n.c.	n.c.	0.002	1.42
	**MMP3**	4314	n.c.	n.c.	n.c.	n.c.	0.035	1.32	n.c.	n.c.
	**ADAM9**	8754	n.c.	n.c.	0.037	1.32	n.c.	n.c.	n.c.	n.c.
	**ADAMTSL2**	9719	0.039	0.81	n.c.	n.c.	n.c.	n.c.	n.c.	n.c.
Adhesionmolecules	**CD47**	961	0.024	1.43	n.c.	n.c.	n.c.	n.c.	n.c.	n.c.
	**ITGAV**	3685	n.c.	n.c.	n.c.	n.c.	0.022	1.33	n.c.	n.c.
	**MCAM**	4162	n.c.	n.c.	n.c.	n.c.	n.c.	n.c.	0.017	1.35
	**CDH5**	1003	0.04	0.71	0.03	0.70	<0.001	0.51	0.011	0.65
	**CD24**	1E+08	0.005	0.73	n.c.	n.c.	<0.001	0.59	<0.001	0.67
	**CD99L2**	83692	n.c.	n.c.	n.c.	n.c.	0.008	0.80	n.c.	n.c.
Signaling molecules	**GDI2**	2665	n.c.	n.c.	n.c.	n.c.	0.008	1.27	n.c.	n.c.
	**FOS**	2353	n.c.	n.c.	0.003	0.72	0.004	0.72	0.001	0.70
	**JUN**	3725	0.014	0.78	0.027	0.80	0.03	0.81	n.c.	n.c.
	**MAPKAPK2**	9261	n.c.	n.c.	0.019	0.75	0.031	0.77	n.c.	n.c.
	**CREBBP**	1387	n.c.	n.c.	0.013	0.75	n.c.	n.c.	n.c.	n.c.
	**MAP4K3**	8491	n.c.	n.c.	0.025	0.80	n.c.	n.c.	n.c.	n.c.
Kinesins	**KIF1B**	23095	n.c.	n.c.	0.004	0.72	n.c.	n.c.	n.c.	n.c.
	**KIF6**	221458	n.c.	n.c.	n.c.	n.c.	0.045	0.80	n.c.	n.c.
	**KIF1C**	10749	n.c.	n.c.	n.c.	n.c.	n.c.	n.c.	0.003	0.69
Membrane proteins	**CAV1**	857	n.c.	n.c.	n.c.	n.c.	0.001	0.76	<0.001	0.70
	**CAV2**	858	n.c.	n.c.	n.c.	n.c.	0.001	0.71	n.c.	n.c.
Binding proteins	**IGFBP5**	3488	0.023	0.81	<0.001	0.66	<0.001	0.65	<0.001	0.61
	**IGFBP3**	3486	n.c.	n.c.	n.c.	n.c.	0.004	0.73	0.009	0.76
ECM proteins	**VCAN**	1462	n.c.	n.c.	0.013	0.75	0.028	0.78	n.c.	n.c.

RE: Relative expression compared to unstimulated MSCs

n.c.: no changes

ECM: Extracellular matrix

### MSCs exposed to human HCC conditioned medium modulated migratory capacity of other cell components of tumor microenvironment

**Figure 4:**
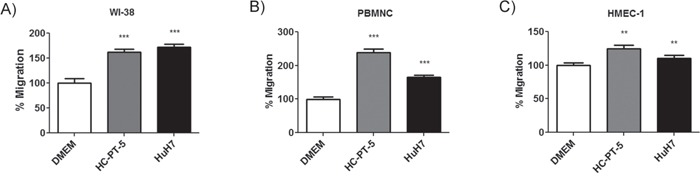
HCC CM-stimulated MSCs modulated migratory capacity of components of tumor microenvironment Chemotaxis assay of fibroblasts **A.**, peripheral blood mononuclear cells **B.,** or endothelial cells **C.,** towards CM of stimulated MSCs with HC-PT-5 (grey bars), with HuH7 (black bars) or unstimulated MSCs (DMEM, white bars). Results are expressed as percentage of control (DMEM) ± SEM of three independent experiments, each performed in quadruplicate. **p<0.01 and ***p<0.001 vs unstimulated MSCs (DMEM, ANOVA and Dunn's post test).

**Figure 5:**
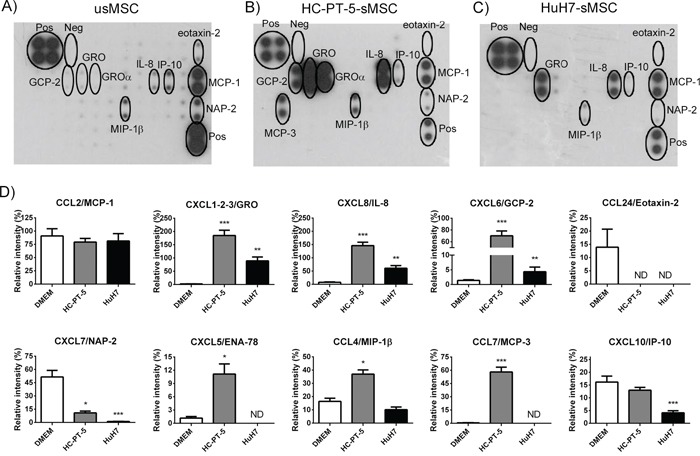
HCC CM modulated MSC chemokine profile. Antibody array of CM from unstimulated MSCs **A.**, HC-PT-5-stimulated MSCs **B.,** or HuH7-stimulated MSCs **C. D.** Quantification of the antibody array signal by densitometry of CM of unstimulated MSCs (DMEM), HC-PT-5-stimulated MSCs (HC-PT-5) or HuH7-stimulated MSCs (HuH7). Results are expressed as relative intensity (%) and positive controls were used to normalize the results. *p<0.05, **p<0.01 and ***p<0.001 vs unstimulated MSCs (DMEM, ANOVA and Dunn's post test). ND: non detectable.

Tumors and their microenvironment establish an extensive cross-talk that involves several cytokines leading to tumor growth and spread. We asked if once MSCs arrive to HCC could change the behavior of the cellular components that form the tumor milieu and if the effect of MSCs could depend of the exposure to the factors present in tumor microenvironment. With this aim, we performed chemotaxis assays of fibroblasts (WI-38), peripheral blood mononuclear cells (PBMNCs) or endothelial cells (HMEC-1) toward conditioned media derived from MSCs stimulated with HuH7 CM or HC-PT-5 CM as wells as unstimulated MSCs as control. Migration assay showed that WI-38 cells, PBMNCs and HMEC-1 cells not only were able to migrate toward MSCs CM but also this migration was increased when CM was obtained from MSCs previously stimulated with HuH7 or HC-PT-5 conditioned media (Figure 4A–4C). In order to identify which factors could be involved in this phenomenon we characterize the chemokine profile of the CM of MSCs stimulated with the CM of HuH7 and HC-PT-5. Protein array shows that the 5 most expressed chemokines in unstimulated MSCs CM were CCL2/MCP-1, CXCL7/NAP-2, CXCL10/IP-10, CCL4/MIP-1β and CCL24/eotaxin-2 while CXCL8/IL-8, CXCL6/GCP-2 and CXCL1-2-3/GRO were moderately expressed (Figure 5A). Interestingly, CM derived from MSCs exposed to HC-PT-5 CM or HuH-7 CM have similar levels of CCL2/MCP-1, higher levels of CXCL1-2-3/GRO, CXCL8/IL-8, CXCL6/GCP-2 and lower levels of CCL24/eotaxin-2 and CXCL7/NAP-2 in comparison of CM of unexposed MSCs (Figure 5A–5D). It should be noted that MSCs exposed to HC-PT-5 CM increased their secretion of CXCL5/ENA-78, CCL4/MIP-1β and CCL7/MCP-3 while MSCs exposed to HuH7 decrease the release of CXCL10/IP-10 (Figure 5D).

### Effect of MSCs stimulated with HCC CM on HCC aggressiveness and survival

**Figure 6:**
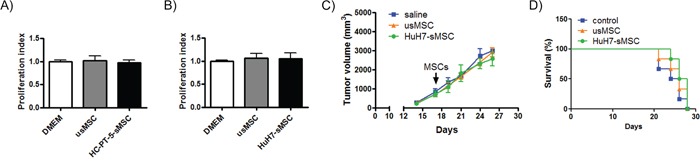
HCC CM-stimulated MSCs did not modify tumor growth or animal survival *In vitro* proliferation of HC-PT-5 **A.,** or HuH7 **B.,** exposed to CM of unstimulated MSCs (usMSC) or CM of MSC stimulated with HC-PT-5 (HC-PT-5-sMSC) or stimulated with HuH7 (HuH7-sMSC). Data represent the average of three experiments. No differences in proliferation were found compared to HCC cells (DMEM). **C.** *In vivo* tumor growth of s.c. HuH7 tumor in nude mice (n=7/group). Seventeen days after tumor inoculation, mice were peritumoral administrated with unstimulated MSCs (usMSC), HuH7-stimulated MSCs (HuH7-sMSC) or vehicle (saline) as control. **D.** Survival of HuH7 tumor-bearing mice (n=7/group) administrated with unstimulated MSCs (usMSC) or HuH7-stimulated MSCs (HuH7-sMSC). No differences in mice survival compared to control mice. Kaplan-Meier, log rank test.

Finally, we decided to evaluate the effect of MSCs stimulated with tumor CM on tumor aggressiveness. For that purpose, we evaluated *in vitro* proliferation of HCC cells with the CM of MSCs stimulated with HuH7 CM o HC-PT-5 CM. In both cases, we did not observe any modification in the proliferation of HuH7 or HC-PT-5 cells (Figure 6A–6B). Next, unstimulated MSCs and MSCs stimulated with HuH7 CM were peritumoral inoculated in HuH7-bearing tumor nude mice and evaluated tumor progression. Consistently, we observed that the 3 experimental groups showed similar tumor growth, indicating that CM of tumor cells did not modify MSC function on tumor growth (Figure 6C). Importantly, animal survival was similar in all the experimental groups (Figure 6D).

## DISCUSSION

Based on their capability to target sites of inflammation and tumors, MSCs were used as cellular vehicles of therapeutic genes against several types of tumors including HCC [15–17]. Several reports indicate that MSCs can promote cancer cell development and progression due a complex crosstalk between MSCs and tumor microenvironment. In fact, Yan *et al.* isolated HCC-associated MSCs from patient's samples that increase tumor proliferation and invasion through the secretion of S1004A [18]. These results indicated that once MSCs home HCC are modulated through a complex interchange of signals. Therefore, it is necessary to understand how MSCs migrate and interact with HCC microenvironment. We and others have demonstrated in experimental models that systemically injected MSCs have preferential tropism toward HCC tumors in response to paracrine factors [15]. In the present work, we demonstrated for the first time that CXCL8/IL-8, CXCL1-2-3/GRO and CCL2/MCP-1 are crucial axes for MSC migration towards HCC. The analysis of CM derived from fresh human samples and from tumor xenografts showed a conserved pattern of cytokines and demonstrated high levels of these cytokines. Moreover, blockage or knockdown of these axes decreased MSC migration towards HCC CM. In our previous work, we found that MSCs showed an enhanced *in vitro* migration toward HCC in comparison with healthy or fibrotic livers [14]. Similarly, MSCs showed higher migration levels to HCC CM when compared with HSCs (LX-2), endothelial cells (HMEC-1) or fibroblast (WI-38) CM [14]. In line with this, in the present work we found that MSCs preferentially migrated *in vitro* toward CM derived from patient tumors in comparison to those derived from adjacent tissue. It is worth noting that CXCL8/IL-8, CCL2/MCP-1 and CXCL1-2-3/GRO were also found in the CM of AT-PT-12 and HSCs (LX-2). However, these CM induced a minor chemotaxis of MSCs. Furthermore, CM from AT-PT-7 showed the lowest CXCL8/IL-8 and CXCL1-2-3/GRO levels and did not induce MSC migration. Simultaneously blockage of CXCR1 and CXCR2 inhibited ~50% MSC migration. These results support a possible role of these cytokines in the MSC migration toward HCC. In line with this, a similar effect was observed in neutrophil migration towards CXCL8/IL-8 and other chemokines such as CCL2/MCP-1, CCL8/MCP-2, CCL7/MCP-3 or CXCL12/SDF-1 [19]. Recently, we have also demonstrated that the AMF/AMFR axis is, at least in part, responsible for MSC migration to HCC [12]. Interestingly, we observed that simultaneous blockage of CXCR1, CXCR2 and AMF inhibited >60% MSC migration. In this line, Lejmi *et al*. reported that CCL15/MIP-1 δ and CCL20/MIP-3α produced by HuH7 cell line are also implicated in MSC migration toward HCC [20]. These results are in agreement with the current knowledge of MSC tropism indicating that MSCs migrate to each tumor by a complex combination of different signals [9]. This effect can occur likely in a cooperative way allowing the cell to use its maximum migratory potential. Taking together these findings could serve to enhance MSC migration towards HCC microenvironment using MSCs genetically modified to overexpress chemokine's receptors.

As we presume that cytokine stimulation on MSCs not only functions as chemotactic axis but also induces changes in MSCs behavior, we evaluated the effect of tumor CM stimulation on MSC recruitment potential. In this study, we demonstrated that HCC CM stimulation of MSCs not only increased the migratory response of MSCs but also induced changes in their gene expression pattern. We observed that CM-stimulated MSCs differentially expressed genes involved in several cellular processes including re-organization of the cytoskeleton, cell adhesion and extracellular matrix remodeling, required for migration and invasion. It should be noted that modulation of genes related with the AMF/AMFR chemotactic axis was observed after MSC exposure to the HCC CM. In particular, an increase in the mRNA level of AMFR regardless the HCC CM used was observed. Moreover, modulation of other genes related with the stimulation of this axis was observed including caveolin-1, caveolin-2, GDI-2, IGFBP3 and MMP-3 [12]. Together, these cell mediators could play a role in a positive loop to enhance MSC migration. In addition to the AMF/AMFR related genes, the modulation of additional cytokines, chemokines and growth factors may indicate that MSC stimulation with HCC CM could enhance the migratory response by autocrine loop stimulation or by paracrine recruitment of other MSCs or cellular components to the tumor milieu. In fact, CM generated by HCC-stimulated MSCs showed an increased capability to recruit fibroblasts, PBMNC and endothelial cells, probably due to an increase in chemokines such as CXCL8/IL-8, CXCL1-2-3/GRO and CXCL6/GCP-2.

Despite researchers efforts to elucidate the impact of MSCs on HCC growth and progression, there are still some concerns about their safety. Even founding that HCC-stimulated MSCs increased secretion of chemokines and the capability to recruit fibroblasts, PBMNC and endothelial cells, we did not observe any induction on tumor growth nor *in vitro* nor *in vivo* and the survival of tumor-bearing mice was similar for those inoculated or not with CM-stimulated MSCs. Besides the effect of MSCs in HCC growth and metastasis remains controversial, we and others have previously demonstrated the safety of inoculated MSCs in HCC models [12, 14, 15].

Taking together our results suggest that MSC migration towards HCC occurs in response to chemotactic axis that works in a cooperative way including CXCL8/IL-8, CXCL1-2-3/GRO, CCL2/MCP-1 and AMF. In addition, the factors secreted by the HCC modulate the MSCs chemotactic potential and gene profile that in turn promote their recruitment. Moreover, HCC-stimulated MSCs could enhance the recruitment of other cells to the tumor milieu, probably through the secretion of chemokines such as CXCL8/IL-8, CXCL1-2-3/GRO and CXCL6/GCP-2. Considering that HCC-stimulated MSCs did not modify tumor growth, these cells can be considered for future HCC treatments, although methods of enhancing their recruitment need to be further investigated.

## MATERIALS AND METHODS

### Ethics statement

Animals were maintained at our Animal Resource Facilities (School of Biomedical Sciences, Austral University) in accordance with the experimental ethical committee and the NIH guidelines on the ethical use of animals. The “Animal Care Committee” from School of Biomedical Sciences, Austral University, approved the experimental protocol.

MSCs and peripheral blood mononuclear cells (PBMNCs) were obtained from bone marrow and whole blood of healthy donors after informed consent and protocol was approved by the “Institutional Evaluation Committee” (CIE) from School of Biomedical Sciences, Austral University (Protocol No. 12-019).

### Cell lines

Human HCC cell line HuH7 were kindly provided by Prof. Jesus Prieto (CIMA, University of Navarra, Pamplona, Spain) [21]. LX-2 cell line (human HSCs generated by spontaneous immortalization in low serum conditions) was kindly provided by Dr. Scott Friedman (Division of Liver Diseases, Mount Sinai School of Medicine, New York, USA). PT-67 retroviral packaging cells were obtained from Clontech. Human microvascular endothelial cells (HMEC-1) were from CDC (Centers for Disease Control, Atlanta, GA, USA) and WI-38 (human fibroblast cell line) was obtained from the American Type Culture Collection. Cell lines were cultured in complete DMEM (2 μM glutamine, 100 U/ml penicillin, 100 mg/ml streptomycin) and 10% heat-inactivated fetal bovine serum (FBS). Primary culture of HCC cells (HC-PT-5) was previously generated in our laboratory and was cultured up to 8 passages in 70% DMEM/30% F12 culture medium (Invitrogen/Life Technologies) supplemented with 2 μM glutamine, 100 U/ml penicillin, 100 mg/ml streptomycin and 10% FBS [14]. Peripheral blood mononuclear cells (PBMC) were isolated by Ficoll-Paque gradient resuspended in complete DMEM without FBS and used for experiments.

### Isolation of BM-MSCs

MSCs were obtained from bone marrow of healthy donors (Hospital Naval Pedro Mallo, Buenos Aires, Argentina) and were characterized according to the International Society for Cellular Therapy (ISCT) guidelines as described previously [14].

### Conditioned medium

Tumoral and adjacent tissues from patients with HCC were obtained at the time of surgical resection or liver transplantation at our institution (Austral Universitary Hospital, Pilar, Buenos Aires, Argentina). The project was approved by the “Institutional Evaluation Committee” (CIE) from School of Biomedical Sciences, Austral University (Protocol No. 11-007) and written informed consent was obtained from all patients.

For the generation of mice xenograft, HuH7 cells (2 × 10^6^) or HC-PT-5 cells (5 × 10^6^) were inoculated subcutaneously (s.c.) into the right flank of nude mice and dissected when tumors reached 200 mm^3^ in size approximately. In all cases the tissues were minced into pieces smaller than 1 mm^3^ and transferred to a 24-well tissue culture plate (6 fragments/well) with 500 μl of complete DMEM without FBS. After 18 h, tumor CM were harvested and stored at −80°C until use.

For MSC CM 1×10^6^cells were plated in 100 mm cell Petri dishes. After 24 h MSCs were washed with PBS and cultured overnight (O.N.) with DMEM medium without FBS. Then MSCs were washed again with PBS and then stimulated with the tumoral CM or unstimulated (DMEM medium without FBS) for 18 h. Finally, cells were washed with PBS and cultured with complete DMEM without FBS and 18 h later, MSC CM were harvested and stored at −80°C until use.

For LX-2 CM, cells were cultured until reached 90% confluence. Then cells were washed with PBS and cultured with complete DMEM without FBS. Eighteen hours later conditioned medium was harvested and stored at −80°C until use.

### Cytokine and chemokine antibody array

The presence of soluble factors in the CM were detected using the RayBio Human Chemokine Antibody Array 1 (Cat# AAH-CHE-1) for tumoral CM and RayBio Human Cytokine Antibody Array 5 (Cat# AAH-CYT-5) for MSC CM (Ray Biotech, Inc.) according to the manufacturer's protocol. The intensities of signals were quantified by densitometry with ImageJ software (National Institute of Health, USA) and positive controls were used to normalize the results from different membranes.

### Gene knockdown with shRNA

Retroviral plasmids (pGFP-V-RS) containing two CXCR1 or CXCR2 specific or scrambled (SCR) negative control shRNAs were prepared as described by the manufacturer's protocol (Origene). The sequences of shRNA were as follows: CGTGTTACCTCCTACACTTCTTCGTCTGT (CXCR1, GI348627), ATCCTGCCTCACACCTTTGGCTTCATCGT (CXCR1, GI348628), TGGTCTCACTCCTGAAGGAAGTCAACTTC (CXCR2, GI348622), AAGGACCGTCTACTCATCCAATGTTAGCC (CXCR2, GI348622), and GCACTACCAGAGCTAACTCAGATCGTACT (scrambled). To produce retroviruses, PT-67 retroviral packaging cells (Clontech) were transfected with retroviral expression plasmids using Lipofectamine 2000 (Invitrogen) and incubated for 48 h. The culture media containing the retroviruses were collected and centrifuged at 2000 × g for 5 min. Then 7.5 × 10^4^ MSCs were seeded in 6-wells plates the night before and cultured with the viral stock and 4 μg/ml polybrene. Medium was replaced 24 h after transduction and migration assay performed 72 h later.

### Gene knockdown with siRNA

Proliferating HuH7 cells (3 × 10^5^) were seeded into 6-well plates and transfected with Trilencer-27 human siRNA against CXCL8/IL-8 (SR302384, Origene), CCL2/MCP-1 (SR304273, Origene) or universal scrambled negative control (SCR) with Lipofectamine 2000 (Invitrogen) according to the manufacturer's instructions. For CM production, 96 h later cells were washed with PBS and cultured with complete DMEM without FBS. Eighteen hours later CM was harvested and stored at −80°C until use.

### *In vitro* migration assays

*In vitro* migration was performed using a 48-Transwell microchemotaxis Boyden Chamber unit (Neuroprobe, Inc.) as previously described [14]. MSCs, WI-38 cells, HMEC-1 cells and PBMNCs (1.2 × 10^3^ cells/well) were placed in the upper chamber and CM was applied to the lower chamber of the transwell unit. For blocking experiments, CM or MSCs were preincubated for 60 min with anti-CCL2/MCP-1 (5 μg/μl), anti-HGF (5 μg/μl), anti-CXCR1 (5 μg/μl), anti-CXCR2 (5 μg/μl), anti-AMF (5 μg/μl) or isotype control IgG (5 μg/μl). For CM stimulation, MSCs were incubated O.N. with the CM or DMEM without FBS as control. The system was left for 4 hours at 37°C in a 5% CO_2_ humidified atmosphere. Cells attached to the lower side of the membrane were fixed in 2% formaldehyde, stained with 4′,6-diamidino-2-phenylindole dihydrochloride (DAPI, Sigma-Aldrich) and counted using fluorescent-field microscopy at 100X. Captured images from three representative visual fields were analyzed using CellProfiler software (www.cellprofiler.com), and the mean number of cells/field ± SEM was calculated.

### Gene expression profiling and data processing

#### Sample preparation

MSCs were plated at a density of 5 × 10^4^ cells/cm^2^ in 60 mm cell Petri dishes 1 day prior to assay. Then MSCs were washed with PBS and cultured O.N. with DMEM medium without FBS. Finally MSC were washed again with PBS and then stimulated with the tumoral CM or unstimulated (DMEM medium without FBS). After 24 h, total RNA was isolated using the TRIzol reagent (Invitrogen) followed by purification using RNeasy mini kit columns (Qiagen) according to the manufacturer′s instructions. Then 10 μg of each RNA was subjected to enzymatic digestion with 1X buffer of DNAase, 2 U DNAse I (AmbionInc), 40 U of ribonuclease inhibitor (RNAaseOUT) and incubated for 30 minutes at 37°C. At the end of the incubation time, RNA was purified with the E.Z.N.A.® Total RNA Kit I (Omega Bio-Tek), following the supplier's protocol. Finally, the RNA was eluted from the column with 20 μl of water and the quantity and purity of RNA determined using UV spectrophotometry (NanoDrop Technologies) and its integrity was verified by 1% agarose gel electrophoresis.

#### Microarray labeling and hybridization

The slides used for hybridization were 48.5K Human Exonic Evidence Based Oligonucleotide (HEEBO) from Microarray Inc. (Nashville, TN, USA) based on a probe set designed by Illumina (San Diego, http://www.illumina.com) and Stanford University. A detailed description of these arrays can be found (http://www.microarray.org/sfgf/heebo.do). Amplification and labeling for the regular microarray strategy were done using the SuperScript Indirect RNA Amplification System (Invitrogen), following the supplier's instructions. An indirect design was used for the hybridization of the microarray, where each aRNA from cells with and without treatment were labeled with Alexa 647, was hybridized against an aRNA from a Universal Reference labeled with Alexa 555 (Human Universal Reference, Clontech). Before hybridization, the slides were pre-blocked with 5X SSC, 0.1% SDS and 0.1% BSA. Then, 60 picomoles of each fluorescent-labeled probe were mixed with 1X hybridization solution (5X SSC, 50% formamide, 0.1% SDS, and 0.01% salmon sperm DNA) and heated at 95°C for 2 min. After hybridization for 16 h at 42°C, the slides were washed once in 2X SSC and 0.1% SDS for 5 min at 42°C, washed in 2X SSC and 0.1% SDS for 5 min at room temperature, and washed twice with 0.1X SSC for 1 min. Finally, the slides were centrifugally dried and scanned in a ScanArrayGx (PerkinElmer).

#### Data analysis

The signal intensity of the slides was quantified using the SpotReaderSoftware (Niles Scientific, USA). The “orange pack 1” standard and an adaptive elliptical circle were used to grid the image. The artifacts inside the glass were eliminated manually and not used in the analysis.

### Reverse transcription-polymerase chain reaction (RT-PCR)

Total RNA isolated as described before (4 μg) was reverse transcribed with 200 U of SuperScript II Reverse Transcriptase (Invitrogen) using 500 ng of Oligo (dT) primers. cDNAs were subjected to real-time polymerase chain reaction (qPCR) (Stratagene Mx3005p, Stratagene). For qRT-PCR, the mRNA levels of the different genes were quantified by SYBR® Green (Invitrogen), using the following primers: CXCR1 forward 5′-TAAGTGGAGCCCCGTGGGG-3′ and reverse 5′-TTTGGATGGTAAGCCTGGC-3′; CXCR2 forward 5′-TTTTCCGCCAGGCTTACCAT-3′ and reverse 5′-AACACCATCCGCCATTTTGC-3′; CXCL8/IL-8 forward 5′-ATGACTTCCAAGCTGGCCGTGGCT-3′ and reverse 5′-TCTCAGCCCTCTTCAAAAACTTCTC-3′; CCL2/MCP-1 forward 5′-CCACCTGGACAAGCAAACCCAA-3′ and reverse 5′-AACAGGGTGTCTGGGGAAAGC-3′; AMFR forward 5′-ACAAGATGTGGGCCTTGCAAGA-3′ and reverse 5′-AAAACGCAGTGCTCCCAGGATA-3′; CXCL1/GRO-1 forward 5′-TGAAGGCAGGGGAATGTATGTG-3′ and reverse 5′-AGCCCCTTTGTTCTAAGCCA-3′; CXCL6/GCP-2 forward 5′-TCAGCGGAGCAGTTTTCTGGA-3′ and reverse 5′-TTCAGGGAGAAGCGTAGGCTTT-3′; PDGFR-A forward 5′-ACAAGCTGTATCACTGCCTTCG-3′ and reverse 5′-AAAACATGAACAGGGGCATTCG-3′; ADAM9 forward 5′-TACCAACCTATGCAGCCAAGCAAC-3′ and reverse 5′-GCAGGACGGGCAGGAATTAAGTTT-3′; CXCL12/SDF-1 forward 5′-GCCATGGAGGCACTAACAAACT-3′ and reverse 5′-TTGGAACCTGAAACCCTGCTGT-3′; IGFBP3 forward 5′-ACTGTGGCCATGACTGAGGAAA-3′ and reverse 5′-AGAGTCTCCCTGAGCCTGACTT-3′. PCR amplifications were carried out using a cycle of 95°C for 10 min and 45 cycles under the following parameters: 95°C for 30 seconds, 58°C for 30 seconds, 72°C for 1 min. At the end of PCR reaction, the temperature was increased from 60°C to 95°C at a rate of 2°C/min, and the fluorescence was measured every 15 seconds to construct the melting curve. Values were normalized to levels of glyceraldehyde-3-phosphate dehydrogenase (GAPDH; used as housekeeping) transcript (forward 5′-CATCTCTGCCCCCTCTGCTG-3′ and reverse 5′-GCCTGCTTCACCACCTTCTTG-3′). Data were processed by the ΔΔct method. The relative amount of the PCR product amplified from untreated cells was set as 1. A non-template control (NTC) was run in every assay, and all determinations were performed as triplicates in three separated experiments.

### Proliferation assays

Cell proliferation was evaluated by [^3^H]-thymidine incorporation assay. Briefly, HCC cells were seeded in 96-well culture tissue plates at 3 × 10^4^ cells/cm^2^ density for 1 day prior to the assay. Then cells were cultured with DMEM (control) or CM obtained from stimulated MSCs with the tumoral CM (HuH7 or HC-PT-5) or control unstimulated MSCs (culture in DMEM) for 48 h followed by a pulse of [^3^H]-thymidine 18 h before the end of the experiment. Finally, [^3^H]-thymidine incorporation was measured in a scintillation counter. Each sample was assayed in sextuplicates and normalized to DMEM as control.

### Mice and *in vivo* experiments

Six- to eight-week-old male nude (Nu/Nu) mice were purchased from CNEA (Comisión Nacional de Energía Atómica, Ezeiza, Buenos Aires, Argentina). Subcutaneously HuH7 tumors (2 × 10^6^ cells) were established and 17 days later MSCs or stimulated MSCs with CM of HuH7 were peritumoral injected. Tumor growth was assessed by calliper measurement, and tumor volume (mm^3^) was calculated by the formula π/6 x larger diameter x (smaller diameter)^2^.

### Statistical analyses

One-way analysis of variance, Kruskal-Wallis test, t-test or Kaplan-Meier log rank (GraphPad Software) were used to statistically examine the differences between groups. Differences with p values lower than 0.05 were considered as statistically significant.
